# Ross 708 broiler small intestine morphology and immunity improvements in response to in ovo Marek's Disease vaccine administration alone or in conjunction with in ovo and dietary supplemental calcifediol

**DOI:** 10.1016/j.psj.2024.104098

**Published:** 2024-07-14

**Authors:** S.A. Fatemi, A.W. Levy, E.D. Peebles

**Affiliations:** ⁎ Department of Poultry Science, Mississippi State University, MS 39762, USA; † DSM Nutritional products, Parsippany, NJ, 07054, USA

**Keywords:** calcifediol, small intestine morphology, α-1-acid glycoprotein, in ovo administration, Marek's Disease vaccine

## Abstract

Investigations were performed to determine the systemic immune and small intestine (**SI**) morphological responses of Ross 708 broilers to the Marek's Disease vaccine (MDV) administered alone or in conjunction with the in ovo and dietary administration of calcifediol (25OHD_3_). Live embryonated hatching eggs were assigned at random to 3 in ovo treatments at 18 d of incubation. Pre-specified in ovo treatments were: commercial MDV-alone-injected (50 µL) or commercial MDV containing 1.2 (MDV+25OHD_3_-1.2) or 2.4 (MDV+25OHD_3_-2.4) μg of 25OHD_3_. A noninjected control treatment was also included. For the growing phase, broilers received a commercial diet containing 250 IU of vitamin D_3_ /kg (control) or a commercial diet supplemented with 2,760 IU of 25OHD_3_ /kg (Hy-D diet). For determination of serum IgG, nitric oxide, and α-1-acid glycoprotein (AGP) at 14 and 40 d of age (doa), blood was collected from 1 bird per pen (48 total). In the duodenum, jejunum, and ileum of the same bird, villus length (VL), crypt depth (CD), VL to CD ratio (VCR), and villus surface area were also determined. There were no significant dietary x in ovo treatment interactions for any of the variables examined. However, birds fed Hy-D diets had lower serum AGP levels at 14 doa when compared to those fed un-supplemented commercial diets. Additionally, at 40 doa, birds in the MDV+25OHD_3_-1.2 and MDV+25OHD_3_-2.4 treatments experienced a decrease in serum AGP in comparison to those belonging to the noninjected and MDV-alone treatment groups. A higher jejunal VCR was observed at 14 and 40 doa in birds that belonged to the MDV+25OHD_3_-1.2 treatment when compared to those in the noninjected and MDV-alone treatment groups, and dietary Hy-D increased the VL of the duodenum and jejunum in birds at 14 and 40 doa when compared to those fed the commercial diet. In conclusion, both dietary or in ovo administration of 25OHD_3_ lowered inflammatory reactions and improved the SI morphology of broilers that were in ovo*-*injected with the MDV.

## INTRODUCTION

It has been more than 3 decades since in ovo technology first emerged for vaccine delivery in broiler hatcheries (Ricks et al., 1999; Peebles, 2018). Prior to that time, the Inovoject machine, manufactured by Embrex, Inc., was the first automated system to be introduced in the US (Ricks et al., 1999). The effects of the in ovo injection of various materials including vaccines and essential nutrients have been previously investigated and subsequently resulted in not only an increase in perinatal development, but also improvements in the posthatch performances and gut health of poultry (Peebles, 2018; Das et al., 2021; Pandey et al., 2021; Razib et al., 2021). Currently, the Marek's disease vaccine (**MDV,** turkey herpesvirus) is the most common vaccine that has been used in broiler hatcheries in the US (Peebles, 2018). The in ovo administration of the MDV at 18 d of incubation (**doi**) in the amnion has been shown to provide a minimum of 90% protection against Marek`s disease (Williams, 2007, Williams, 2011), and to increase villus length (**VL**) to crypt depth (**CD**) ratio (**VCR,** Peebles et al., 2017, 2019), as well as the expression of genes associated with humoral immunity in newly-hatched broilers (Gimeno et al., 2018).

In chickens, vitamin D can be absorbed in cooperation with ultraviolet light and 7-dehydrocholesterol in the skin. It can also be provided as a supplement in the feed. Vitamin D is commonly supplied in poultry diets as cholecalciferol (**D_3_**), and is mainly absorbed in the jejunum (De Matos, 2008). Bile salts facilitate the absorption of dietary lipophilic compounds including D_3_ and calcifediol (**25OHD_3_**). Two sequential hydroxylation steps function in the conversion of D_3_ to its active form. The first of these steps takes place in liver microsomes and mitochondria, where D_3_ is converted to 25OHD_3_ through 25-hydroxylase activity (Norman, 1987; Fotenhauer and Shubrook, 2017). Also, the conversion of D_3_ to 25OHD_3_ takes place at a lower rate in the intestine or kidney (Norman, 1987; Shanmugasundaram and Selvaraj, 2012). Subsequently, 25OHD_3_ is transported via vitamin D binding proteins to the kidneys, where 25OHD_3_ is hydroxylated by 1α-hydroxylase to 1,25-dihydroxycholecalciferol (**1,25(OH)_2_ D_3_**), the active form of vitamin D (Henry, 1980; De Matos, 2008). As compared to D_3,_ the dietary or in ovo administration of 25OHD_3_ has been shown to improve overall hatchling quality (Fatemi et al., 2020a,b), as well as the bone quality (Fritts and Waldroup, 2003; Bello et al., 2014; Yair et al., 2015), performance (Soares et al., 1995; Yarger et al., 1995; Fatemi et al., 2021a,b,d), breast and leg meat yield (Yarger et al., 1995; Vignale et al., 2015; Fatemi et al., 2021a,b,d), muscle development (Hutton et al., 2014; Vignale et al., 2015; Fatemi, 2016; Chen et al., 2021), innate and adaptive immunity (Morris and Selvaraj, 2014; Morris et al., 2014; Morris et al., 2015; Shojadoost et al., 2015; Fatemi, 2016; Shanmugasundaram et al., 2019; Chen et al., 2021; Fatemi et al., 2021a, c; Fatemi et al., 2022b), antioxidant activity (Nong et al., 2023), and small intestine (**SI**) morphology (Chou et al., 2009; Ding et al., 2011; Wang et al., 2021; Fatemi et al., 2021c, 2022b) of broilers reared under commercial conditions or when subjected to a challenging environment. It is suggested that these improvements in response to supplemental 25OHD_3_ can be attributed to the fact that 25OHD_3_ has a longer half-life (3 wk, Hollis and Wagner, 2013) than D_3_ (15 h, Smith and Goodman, 1971), is stored to a greater extent in muscle tissue (Burild et al., 2016), and has the ability to promote an increase in calcium (**Ca**) and phosphorus (**P**) absorption in the upper portion of the SI (Bar et al., 1980).

The effects of the in ovo administration of 25OHD_3_ in combination with the **MDV** have been previously investigated on the hatch, live performance, and meat yield of broilers. These results showed that in comparison to noninjected or MDV-alone-injected control treatments, 1.2 and 2.4 μg of 25OHD_3_ increased the breast and leg meat yield and BW and BW gain (**BWG**) of broilers (Fatemi et al., 2024b). In addition, the genes linked to vitamin D activity and humoral immunity were up-regulated in broiler hatchlings in response to 1.2 and 2.4 μg of 25OHD_3_ (Fatemi et al., 2024a). Furthermore, dietary 25OHD_3_ at 2,760 IU/ kg feed improved the performance and meat yield of broilers throughout the rearing period in comparison to those fed the commercial diet. Thus, both 25OHD_3_ sources are potent enhancers of the posthatch performance and meat yield of broilers that had received an in ovo injection of the MDV. The promising performance and meat yield results observed in broilers in response to the in ovo and dietary administration of 25OHD_3_ in conjunction with MDV could be related to an enhancement in their small intestine (**SI**) morphology and inflammatory responses. An increase in VL is linked to a greater absorption area, and a deeper CD indicates that the villi in the SI mucosa are atrophied and that their absorptive capacity is reduced (Zhang et al., 2005). Furthermore, a decrease in chronic inflammation in response to dietary or in ovo of 25OHD_3_ (Morris et al., 2015; Shanmugasundaram et al., 2019; Fatemi et al., 2021c;b) has been shown to promote performance (Shanmugasundaram et al., 2019; Morris et al., 2015; Fatemi et al., 2021d) and beast meat yield (Fatemi et al., 2021d) of poultry. Thus, an improvement in SI morphology variables as well as a reduction in chronic inflammatory responses can be beneficial to broiler growth and production. Although the strong relationship between SI morphology and immunity with the posthatch performance of broilers in response to in ovo and dietary sources of 25OHD_3_ was previously reported, the aforementioned effects on SI morphology and immunity were not tested when 25OHD_3_ sources were administered along with the in ovo administration of the MDV. It is hypothesized that the in ovo and dietary sources of 25OHD_3_ provided separately or together can improve SI morphology and decrease inflammation when administered along with an in ovo injection of the MDV. Therefore, the objectives in the current study were to determine the impacts of the dietary and in ovo application of various levels of 25OHD_3_ on the SI morphology and immunity of Ross 708 broilers that received an in ovo injection of the MDV.

## MATERIALS AND METHODS

### Experimental Design and Treatment Layout

The Institutional Animal Care and Use Committee of Mississippi State University (Protocol IACUC-20-248) approved the experimental procedures of this study. After fertile eggs were collected from 35-wk-old commercial Ross 708 broiler breeder hens, they were stored under commercial conditions (12.8 °C and 10.4 °C dry and wet bulb temperatures, respectively) for 24 h (Lindsey et al., 2023; Mousstaaid et al., 2023a). Prior to set, eggs were selected as described by Sokale et al. (2017) and a total of 1,920 eggs (mean weight = 60.1 ± 0.91 g) were subsequently set and incubated in a single-stage setter/ hatcher incubator (Chick Master Incubator Company, Medina, OH) under standard conditions (37.5°C dry bulb and 29.0°C wet bulb temperatures, respectively) according to the procedure described by Fatemi et al. (2020b). Forty eggs were assigned to each of 4 pre-assigned treatment groups on each of 12 incubator tray levels (blocks). All in ovo injection solutions, using the form and source of 25OHD_3_ (ROVIMIX Hy-D 1.25%; DSM Nutritional Products Inc., Parsippany, NJ) as described by Fatemi et al. (2020a,b), were prepared and injected according to the procedures of Fatemi et al., 2023, Fatemi et al., 2024a. A 50 µL volume of each pre-specified treatment were injected into the amnion at 18 doi using a Zoetis Inovoject m (Zoetis Animal Health, Research Triangle Park, NC) multi-egg injection machine. The 3 in ovo injection treatments were pre-specified as: commercial MDV-alone-injected or commercial MDV containing either 1.2 (**MDV + 25OHD_3_-1.2**) or 2.4 (**MDV + 25OHD_3_-2.4**) μg of 25OHD_3_. A noninjected control treatment was also included.

Hatch was pulled at 502 h of incubation (21 doi). At hatch, 13 male broilers were assigned to each of 48 floor pens containing used litter at a 0.062 m^2^/bird stocking density according to the procedure described by Fatemi et al. (2024b). The 2 dietary treatments throughout the growing phase were: 1) a control commercial diet, which contained 250 IU of D_3_ /kg of feed (control); or 2) the control commercial diet which also contained 69 μg (equivalent to 2,760 IU) of 25OHD_3_ /kg of feed (**Hy-D** diet). There were a total of 8 treatment groups (2 dietary treatments x 4 in ovo treatments) in the experimental design, with 6 replicate pens being represented in each of the 8 treatment groups (48 total pens). Fresh water and feed as pellets were provided *ad libitum* throughout the growing phase. A Mississippi State University basal corn-soybean diet was used this study and was formulated according to Ross 708 commercial guidelines (Aviagen, 2015; Fatemi et al., 2021a,b,d; Fatemi et al., 2024b). The actual value for the level of the D_3_ in the diets ranged from 80 to 110% of formulated values, and the actual value for the 25OHD_3_ levels in the Hy-D treatment diets ranged from 85 to 101% of formulated values (Fatemi et al., 2024b).

### Somatic Characteristic and Immunological Assessments

At 14 and 40 d of age (**doa**), one bird from each of the 48 floor pens was randomly selected, individually weighed, necropsied, and the weights of their bursa and spleens were determined as a percentage of BW. From the same birds that were selected for organ sampling at 14 and 40 doa, chorioallantoic vasculature blood samples were collected and 1 mL of serum from each sample was extracted for immunological assay (Mousstaaid et al., 2022a,b; 2023b). Serum α-1-acid glycoprotien (**AGP**) concentration was measured in accordance to the method provided by Kaab et al. (2018) and Fatemi et al. (2021a). Serum nitric oxide (**NO**) concentration was evaluated based on the procedure as described by Bowen et al. (2007) and Mousstaaid et al. (2022b). Serum IgG concentration was determined according the procedure described by Fatemi et al. (2021c). At an optical density (**OD**) of 450 nm, all immunological variables were measured with a SpectraMax M5 Microplate Reader (Molecular Devices, San Jose, CA).

### Small Intestine Morphology

At 14 and 40 doa, intestinal samples (5 cm in length) from the same 48 sample birds, were excised from the middle segment of the duodenum, jejunum, and ileum and fixed in 10% formalin with 6 replicates per treatment combination (in ovo x dietary treatments). The SI histomorphological slides were prepared according to the procedure of Wang et al. (2015). Villus length, villus width (**VW**) and CD were measured according to the method described by Nain et al. (2012) and Fatemi et al. (2021c,a). The VCR values were calculated by dividing the VL by the CD. In addition, the surface area for all sections was estimated using the following formula specified by Nain et al. (2012): Villus surface area (**VSA**) = 2π × (average VW/2) × VL, where VW is the mean of 6 measurements per bird (2 width measurements from 3 sample sections on each slide).

Finally, variable means within each of the 3 intestinal regions for each bird were statistically analyzed.

### Statistical Analysis

For all variables that were tested in this study, the experimental unit was the floor pen and the experimental design was a randomized complete block. All data were analyzed using a 2-way ANOVA with a 4×2 factorial arrangement of treatments to test for the main and interactive effects of the 4 in ovo injection treatments and 2 dietary treatments.

Data analysis was performed using the General linear mixed models (PROC GLIMMIX) of SAS 9.4© (SAS Institute, 2013), and differences between treatment means were considered as significant at *P* ≤ 0.05. In addition, noticeable trends were deemed at *P* < 0.08. Fisher's protected least significant difference was used for treatment means separations (Steel and Torrie, 1980). The power analysis employed is based on a standard error of 0.8, an α = 0.05, and a standard deviation of 4. This analysis indicated that in order to achieve statistically relevant results with a minimal error rate, 4 replicate groups per treatment, with a maximum of 14 newly hatched chicks per replicate group was needed.

## RESULTS

### Somatic Characteristics

The results for the BW and relative immunity-related organ (bursa and spleen) weights of the individually necropsied birds at 14 and 40 doa are shown in Table 1. There were no significant interactions between treatments (diet x in ovo) for all variables. However, BW was higher in Hy-D-fed birds than in those fed commercial diets at 14 and 40 doa. In addition, birds that were injected with 1.2 or 2.4 μg of 25OHD_3_ had higher spleen weights than those in the MDV-alone-injected treatment at 14 doa.

**Table 1:** Effects of noninjected and *in ovo* injection treatments of Marek's disease vaccine (**MDV**) alone or MDV containing various doses of calcifediol (**25OHD_3_**), and commercial diets or diets supplemented with 2760 IU/kg of 25OHD_3_ on the mean BW, and weights of the bursa and spleen relative to BW in broilers at 14 and 40 d of age (**doa**).

Treatment		BW (g)	Bursa (%)	Spleen (%)
		–––––14 doa–––––		
In ovo injection				
	Noninjected^1^	453	0.235	0.121^ab^
	MDV^2^	479	0.222	0.107^b^
	MDV + 25OHD_3_-1.2^3^	750	0.237	0.138^a^
	MDV + 25OHD_3_-2.4^4^	466	0.236	0.143^a^
	SEM	18.5	0.0216	0.0132
Diet				
	Commercial	445^b^	0.242	0.135
	Hy-D^5^	479^a^	0.223	0.119
	SEM	13.1	0.0153	0.0094

		–––––P-value–––––		
In ovo		0.397	0.884	**0.038**
Diet		**0.014**	0.208	0.099
In ovo x Diet		0.298	0.461	0.781

		–––––40 doa–––––		
In ovo injection				
	Noninjected	2522	0.115	0.153
	MDV	2720	0.120	0.143
	25OHD_3_-1.2	2786	0.110	0.153
	25OHD_3_-2.4	2729	0.112	0.151
	SEM	158.6	0.0104	0.0154
Diet				
	Commercial	2323^b^	0.114	0.141
	Hy-D	3005^a^	0.117	0.159
	SEM	112.2	0.0073	0.0080

		–––––P-value–––––		
In ovo		0.534	0.816	0.912
Diet		**<.0001**	0.693	0.101
In ovo x Diet		0.839	0.419	0.264

a,b Treatment means within the same column within effect with no common superscripts are significantly different (P ≤ 0.05).

1 Embryos that did not receive a solution injection.

2 Received a 50-µL solution volume of the Marek's disease vaccine injected at 18 doi.

3 Embryos injected with the Marek's disease vaccine containing 1.2 μg of 25OHD_3_.

4 Embryos injected with the Marek's disease vaccine containing 2.4 μg of 25OHD_3_^.^

5 A diet supplemented with 2,760 IU/kg 25OHD_3_ throughout the rearing period.

### Immunological Assessments

The results for the immunological measurements (IgG, AGP, and NO) are illustrated in Table 2. At 14 doa, birds fed Hy-D diets had significantly lower serum AGP concentrations than those fed commercial diets. At 40 doa, birds that were injected with either dose of 25OHD_3_ exhibited significantly lower serum AGP concentrations than those in the noninjected and MDV-alone control treatment groups. Additionally, there was a noticeable trend toward a dietary treatment effect (P = 0.078) on serum AGP concentrations at 40 doa, in which birds fed Hy-D diets had numerically lower serum AGP concentrations than those fed commercial diets. Furthermore, there was a noticeable trend for an in ovo x dietary interactive effect (*P* = 0.079) on serum IgG concentrations at 40 doa, in which birds that received the MDV in combination with 25OHD_3_ at the 2.4 µg level had a lower serum IgG concentrations when fed diets supplemented with Hy-D rather than un-supplemented commercial diets (Table 2).

**Table 2:** Effects of noninjected and in ovo injection treatments of Marek's disease vaccine (**MDV**) alone or MDV containing various doses of calcifediol (**25OHD_3_**), and commercial diets or diets supplemented with 2760 IU/kg of 25OHD_3_ on the serum **IgG**, α 1-acid glycoprotein (**AGP**), and nitric oxide (**NO**) concentrations in broilers at 14 and 40 d of age (**doa**).

Treatment		IgG	AGP	NO
		–––––μM–––––		
		–––––14 doa–––––		
In ovo injection				
	Noninjected^1^	3.62	2.26	10.78
	MDV^2^	2.67	1.88	10.83
	MDV + 25OHD_3_-1.2^3^	4.1	2.29	8.28
	MDV + 25OHD_3_-2.4^4^	4.5	2.14	8.41
	SEM	1.004	0.334	2.41
Diet				
	Commercial	3.39	2.42^a^	10.46
	Hy-D^9^	4.05	1.86^b^	8.69
	SEM	0.71	0.236	1.704
		
In ovo		0.315	0.594	0.792
Diet		0.361	**0.024**	0.469
In ovo x Diet		0.477	0.281	0.873

		–––––40 doa–––––		
In ovo injection				
	Noninjected	4.03	4.61^a^	7.82
	MDV	4.79	4.64^a^	7.36
	MDV + 25OHD_3_-1.2	2.63	2.81^b^	6.71
	MDV + 25OHD_3_-2.4	2.27	2.62^b^	6.8
	SEM	1.096	0.677	1.83
Diet				
	Commercial	3.32	4.11	6.39
	Hy-D	3.54	3.23	8
	SEM	0.865	0.516	1.295
Diet x In ovo injection				
	Commercial*Noninjected	3.19	4.96	7.37
	Commercial*MDV	2.14	2.51	6.90
	Commercial*MDV + 25OHD_3_-1.2	1.12	2.98	4.69
	Commercial*MDV + 25OHD_3_-2.4	6.83	6.00	6.61
	Hy-D*Noninjected	4.87	4.26	8.28
	Hy-D*MDV	3.13	3.12	7.82
	Hy-D*MDV + 25OHD_3_-1.2	3.43	2.27	8.74
	Hy-D*MDV + 25OHD_3_-2.4	2.75	3.28	6.98
	SEM	1.903	1.24	2.59
		–––––P-value–––––		
In ovo		0.221	**0.036**	0.922
Diet		0.814	0.078	0.235
In ovo x Diet		0.079	0.315	0.739

a,b Treatment means within the same column within effect with no common superscripts are significantly different (P ≤ 0.05).

1 Embryos that did not receive a solution injection.

2 Received a 50-µL solution volume of the Marek's disease vaccine injected at 18 doi.

3 Embryos injected with the Marek's disease vaccine containing 1.2 μg of 25OHD_3_.

4 Embryos injected with the Marek's disease vaccine containing 2.4 μg of 25OHD_3_. ^5^A diet supplemented with 2,760 IU/kg 25OHD_3_ throughout the rearing period.

### Small Intestine Morphology

No significant interactive effects between the in ovo and dietary treatments on all SI morphology variables were observed at both 14 and 40 doa. Nevertheless, at 14 doa, duodenal VL was higher in Hy-D-fed birds than that in commercial-fed birds. In the duodenum, birds in the MDV + 25OHD_3_-1.2 treatment exhibited a higher VCR than all other in ovo injection treatment groups. There were noticeable numerical trends for both in ovo and dietary treatment main effects for CD in the duodenum at 14 doa, in which duodenal CD tended to be shallower (*P* = 0.064) in the MDV + 25OHD_3_-1.2 treatment than in the noninjected and MDV-alone-injected treatment groups, and birds fed Hy-D diets tended to have a lower (*P* = 0.064) duodenal CD than that those fed commercial diets (Table 3). In the jejunum, VW was higher in Hy-D-fed birds than in those fed commercial diets at 14 doa. Furthermore, jejunal CD was deeper in the noninjected treatment as compared to all the other in ovo injection treatment groups. Also, VCR was greater in the MDV + 25OHD_3_-1.2 and MDV + 25OHD_3_-2.4 treatments than in the noninjected treatment, and a higher VCR in the jejunum was observed in birds in the MDV + 25OHD_3_-2.4 treatment than in birds belonging to the MDV-alone-injected and noninjected treatment groups (Table 3). In the ileum, a lower VW was observed in Hy-D-fed birds in comparison to those fed commercial diets. Additionally, VL in the ileum at 14 doa tended (*P* = 0.070) to be numerically higher in Hy-D-fed birds than in commercial diet-fed birds (Table 3). At 40 doa, VL, VW, and VCR in the duodenum, and VL in the jejunum were greater in birds fed Hy-D diets as compared to those fed commercial diets. Jejunal VCR at 40 doa also tended (*P* = 0.051) to be numerically higher in birds fed Hy-D diets than in the commercial diet-fed birds (Table 4). Jejunal VL was higher in birds belonging to the MDV + 25OHD_3_-1.2 treatment than in the noninjected treatment. Additionally, at 40 doa, jejunal VCR was significantly greater in birds that were injected with either dose of 25OHD_3_ as compared to those in the noninjected treatment, and a significantly higher jejunal VCR was observed in birds belonging to the MDV + 25OHD_3_-1.2 treatment in comparison to those in the MDV-alone-injected and noninjected treatment groups. Furthermore, birds that were injected with the MDV alone or the MDV combined with either dose of 25OHD_3_ experienced a higher ileal VL than in those in the noninjected treatment. A higher ileal VCR at 40 doa was observed in birds belonging to the MDV + 25OHD_3_-2.4 treatment as compared to those in the MDV-alone-injected and noninjected treatment groups. Moreover, birds in the noninjected treatment had a higher VSA than in those in the all the other in ovo injection treatment groups (Table 4).

**Table 3:** Effects of noninjected and in ovo injection treatments of Marek's disease vaccine (**MDV**) alone or MDV containing various doses of calcifediol (**25OHD_3_**), and commercial diets or diets supplemented with 2,760 IU/kg of 25OHD_3_ on the mean small intestine morphology measurements in broilers at 14 d of age (**doa**).

		Duodenum					Jejunum					Ileum				
Treatment		Villus length	Villus width	Crypt depth	VCR	VSA	Villus length	Villus width	Crypt depth	VCR	VSA	Villus length	Villus width	Crypt depth	VCR	VSA
		–––––µm–––––			Length/Depth ratio	mm^2^	–––––µm–––––			Length/Depth ratio	mm^2^	–––––µm–––––			Length/Depth ratio	mm^2^
In ovo injection																
	Noninjected^3^	922	105	132	7.1^b^	0.37	659	100	166^a^	4.3^c^	0.50	455	87	126	3.7	0.31
	MDV^4^	1027	107	135	7.7^b^	0.33	720	102	133^b^	5.5^bc^	0.45	468	97	128	3.8	0.33
	MDV + 25OHD_3_-1.2^5^	968	108	112	8.9^a^	0.35	726	95	108^b^	6.9^ab^	0.42	500	95	119	4.2	0.30
	MDV + 25OHD_3_-2.4^6^	959	107	124	7.8^b^	0.36	723	97	107^b^	7.0^a^	0.44	503	89	122	4.2	0.28
	SEM	52.5	6.1	8.6	0.52	0.025	58.6	7.6	15.8	0.67	0.052	27.5	5.2	8.2	0.30	0.025
Diet																
	Commercial	910^b^	103	120	7.7	0.36	686	91^b^	128	5.9	0.44	463	86^b^	119	3.9	0.30
	Hy-D	1027^a^	111	132	8.0	0.34	728	106^a^	130	5.9	0.47	499	97^a^	129	4.0	0.31
SEM		37.1	3.1	6.1	0.37	0.017	41.4	5.4	11.2	0.47	0.037	19.4	3.7	5.8	0.21	0.018
		–––––P-value–––––														
In ovo		0.264	0.978	0.064	0.015	0.541	0.615	0.796	0.002	0.001	0.529	0.235	0.204	0.719	0.173	0.198
Diet		0.003	0.072	0.064	0.488	0.315	0.312	0.013	0.871	0.902	0.358	0.070	0.004	0.094	0.634	0.445
In ovo x Diet		0.606	0.690	0.690	0.688	0.747	0.409	0.687	0.632	0.700	0.609	0.687	0.053	0.353	0.139	0.598

a-c Treatment means within the same variable column within type of treatment with no common superscript differ significantly (P < 0.05). ^1^Ratio of villus length to crypt depth. ^2^Villus surface area (**VSA**) calculated with average villus length and width = 2π × (width/2) × length.

3 Embryos that did not receive a solution injection.

4 Received a 50-µL solution volume of the Marek's disease vaccine injected at 18 doi.

5 Embryos injected with the Marek's disease vaccine containing 1.2 μg of 25OHD_3_.

6 Embryos injected with the Marek's disease vaccine containing 2.4 μg of 25OHD_3_. ^7^A diet supplemented with 2,760 IU/kg 25OHD_3_ throughout the rearing period.

**Table 4:** Effects of noninjected and in ovo injection treatments of Marek's disease vaccine (**MDV**) alone or MDV containing various doses of calcifediol (**25OHD_3_**), and commercial diets or diets supplemented with 2760 IU/kg of 25OHD_3_ on the mean small intestine morphology measurements in broilers at 40 d of age (**doa**).

		Duodenum					Jejunum					Ileum				
Treatment		Villus length	Villus width	Crypt depth	VCR^1^	VSA^2^	Villus length	Villus width	Crypt depth	VCR	VSA	Villus length	Villus width	Crypt depth	VCR	VSA
		–––––µm–––––			Length/Depth ratio	mm^2^	–––––µm–––––			Length/Depth ratio	mm^2^	–––––µm–––––			Length/Depth ratio	mm^2^
In ovo injection																
	Noninjected^3^	1059	140	144	8.0	0.42	827^b^	127	161	5.6^c^	0.50	467^b^	122	109	4.4^b^	0.46^a^
	MDV^4^	1174	138	145	8.7	0.37	924^ab^	134	149	6.4^bc^	0.47	585^a^	118	125	4.8^b^	0.34^b^
	MDV + 25OHD_3_-1.2^5^	1209	141	127	10.2	0.39	988^a^	132	124	8.3^a^	0.42	590^a^	114	119	5.2^ab^	0.31^b^
	MDV + 25OHD_3_-2.4^6^	1191	127	120	10.3	0.34	883^ab^	116	123	7.4^ab^	0.42	673^a^	109	113	6.2^a^	0.26^b^
	SEM	68.9	9.6	12.9	1.11	0.035	50.6	8.4	19.7	0.711	0.041	54.4	9.6	11.6	0.55	0.055
Diet																
	Commercial	1066^b^	129^b^	141	7.9^b^	0.39	823^b^	122	144	6.5	0.47	563	111	113	5.2	0.33
	Hy-D^7^	1250^a^	144^a^	127	10.7^a^	0.37	979^a^	132	135	7.5	0.45	595	121	120	5.0	0.35
SEM		49.4	6.9	9.2	0.80	0.025	36.0	6.0	14.1	0.49	0.029	39.0	6.9	8.1	0.40	0.040
		–––––P-value–––––														
In ovo		0.147	0.474	0.162	0.137	0.133	0.027	0.171	0.169	0.004	0.230	0.006	0.579	0.541	0.014	0.007
Diet		0.001	0.035	0.138	0.002	0.364	0.001	0.089	0.555	0.051	0.247	0.410	0.147	0.377	0.570	0.670
In ovo x Diet		0.911	0.309	0.953	0.899	0.871	0.955	0.177	0.269	0.444	0.354	0.163	0.101	0.144	0.470	0.915

a-c Treatment means within the same variable column within type of treatment with no common superscript differ significantly (P < 0.05).

1 Ratio of villus length to crypt depth.

2 Villus surface area (**VSA**) calculated with average villus length and width = 2π × (width/2) × length.

3 Embryos that did not receive a solution injection.

4 Received a 50-µL solution volume of the Marek's disease vaccine injected at 18 doi.

5 Embryos injected with the Marek's disease vaccine containing 1.2 μg of 25OHD_3_.

6 Embryos injected with the Marek's disease vaccine containing 2.4 μg of 25OHD_3_^.^

7 A diet supplemented with 2,760 IU/kg 25OHD_3_ throughout the rearing period.

## DISCUSSION

The influences of different in ovo*-*injected 25OHD_3_ doses in conjunction with the MDV on the SI histomorphology and inflammatory reactions of broilers raised under commercial conditions were investigated in this study. The histomorphology results indicated that both the dietary and in ovo sources of 25OHD_3_ had positive influences on the VL, CD, VCR of the SI. Although, when compared to the noninjected control group, MDV-alone-injected birds had a higher jejunal VL at 40 doa and a lower ileal CD at 14 doa, the greater effects observed in response to the 25OHD_3_ sources indicated that 25OHD_3_ was effective when used in combination with the MDV. It is well-observed that rapid morphological development of the SI in broilers occurs late in incubation and during the first wk posthatch (Ding et al. 2011). An increased rate of intestinal epithelial cell turnover is associated with deeper crypts and a heavier SI, which leads to a greater energy and protein demand for gut maintenance (Yason et al., 1987; Yang et al. 2008). In addition, an increase in VL and VCR is associated with more nutrient absorption (Onderci et al., 2006; Fatemi, 2016; Fatemi et al., 2021c; 2022a; Wang et al., 2021), which can subsequently result in increased BW and breast meat yield (Wang et al., 2019; Fatemi et al., b,c,d; Fatemi et al., 2022a). Furthermore, a lower rate of epithelial cell turnover is associated with a shallower CD, which leads to a lower energy requirement in the gut (Yang et al., 2008). It is worth mentioning that gut maintenance energy requirements account for approximately 20% of body energy expenditures (Choct, 2009). A decrease in gut maintenance energy requirements would provide more energy that could be allocated towards production.

Vitamin D_3_ sources have shown a regulatory role in the morphological and functional development of intestinal villi during the prenatal (Ding et al., 2011) and posthatch (Shinki et al., 1991) periods. The influences of dietary D_3_ sources on the SI have been previously reported in broiler embryos and early posthatch hatchlings. More specifically, maternal dietary 25OHD_3_ at 2,760 IU/ kg of feed has proven to increase the VL of broiler embryos at 19 doi and of 1 and 2 doa broiler hatchlings (Ding et al., 2011). In addition, 2,760 IU/kg of supplemental 25OHD_3_ (the same level of inclusion of D_3_) in broiler diets has increased the VL and VCR and reduced the CD at 28 and 35 doa in the duodenum and jejunum (Chou et al., 2009). Also, when layers experienced a high stocking density for 16 wk, VL was increased and CD was decreased in the jejunum at 51 wk of age when they were provided 2,760 IU/kg of supplemental 25OHD_3_ rather than 5000 IU/kg of supplemental D_3_ in their feed (Wang et al., 2021). These results, which are similar to those obtained in this study, indicate that supplemental 25OHD_3_ provided in feed at a level of 2,760 IU/kg, is sufficient to improve the SI morphology of broilers. The impact of the in ovo injection of 25OHD_3_ on SI morphology was also reported for broilers raised under commercial conditions (Fatemi et al., 2021c) or when they were subjected to a coccidiosis infection (Fatemi et al., 2022a). Birds reared in an nonchallenging environment and that were in ovo*-*injected with 2.4 µg of 25OHD_3_ had a greater VL and VCR and a lower CD at 14 and 40 doa as compared to those in diluent-injected or D_3_-injected treatments groups (Fatemi et al., 2021c). Additionally, 2 wk after a coccidiosis infection, duodenal and jejunal SI morphological variables were improved in broilers that had received 1.2 or 2.4 µg of in ovo-injected 25OHD_3_ (Fatemi et al., 2022a). Thus, the in ovo injection of 1.2 and 2.4 µg of 25OHD_3_, alone or in combination with the MDV, can be used to boost the intestinal villus morphology of broilers.

Throughout the rearing period, improvements in the results for SI histomorphology in the upper region of the SI in response to both sources of 25OHD_3_ likely occurred in association with a concurrently higher level of Ca absorption (Bar et al., 1980; De Matos, 2008). Also, due to the function of vitamin D sources that are involved in Ca and P intake, an enhancement in the expression of genes (VDR, 1-αhydroxylase, and 24-hydroxylase) that are involved in vitamin D activity may not only lead to a greater absorption of Ca and P, but also to an improvement in the morphology of the SI. In a study companion to this one, the effects of the same in ovo and dietary treatments on the live performance and meat yield of broilers were investigated (Fatemi et al., 2024b). Results of the study conducted by Fatemi et al. (2024b) revealed that breast meat yield and live performance variables were improved in birds fed Hy-D diets as compared those fed commercial diets throughout the rearing period, and those birds that received MDV + 25OHD_3_-1.2 or MDV + 25OHD_3_-2.4 treatments experienced an increased breast meat yield. In addition, the BW and BW gain of the birds at 14 and 40 doa were improved in comparison to those in MDV-alone-injected or noninjected treatment groups. The improvements in the posthatch performance of broilers in response to the 25OHD_3_ sources could be partially linked to an enhancement of SI morphology. This has been previously reported in birds reared in commercial (Chou et al., 2009; Fatemi et al., 2021b,c) and coccidiosis-challenged (Fatemi et al., 2021d; Fatemi et al., 2022a) conditions.

Fatemi et al. (2022c) suggested that the positive impacts of 25OHD_3_ can be due to a lowering of chronic inflammation and the expression of genes linked to vitamin D activity. During a coccidiosis infection, the expression of 1α-hydroxylase, which converts 25OHD_3_ to its active form, 1,25(OH)_2_ D_3_, is increased. Furthermore, 24-hydroxylase, which converts 25OHD_3_ to the inactive form, 24,25(OH)_2_ D_3_, is decreased in the jejunum. Also, the in ovo administration of 1.2 or 2.4 µg of 25OHD_3_ in conjunction with the MDV has been shown to up-regulate the expression of 1α-hydroxylase and to down-regulate the expression of 24-hydroxylase in comparison to that of birds belonging to a noninjected treatment group (Fatemi et al., 2024a). It is well-documented that an increase in circulating 1,25(OH)_2_ D_3_ levels is associated with an increased intestinal absorption of Ca and P (De Matos, 2008) as well as greater immunological activity (Shanmugasundaram and Selvaraj, 2012; Shojadoost et al., 2015; Shanmugasundaram et al., 2019), which can lead to an improvement in bird performance and meat yield. Therefore, the improvement in morphological variables as a result of the use of in ovo supplemental 25OHD_3_ in combination with the MDV could be linked to a promotion of genes associated with vitamin D activity. However, further research is needed to discover the molecular mechanisms that are involved in these relationships.

The improvement in the SI morphology variables in response to the 25OHD_3_ sources could also be linked to their regulatory role in immunity. A decrease in chronic local inflammatory responses have been shown to be associated with an improvement in SI histomorphology (Fatemi et al., 2022a), bone quality (Fatemi, 2016), performance (Fatemi et al., 2021a,d), and breast meat yield (Fatemi et al., 2021) in broilers. In this study, serum AGP levels, which serve as a chronic inflammatory indicator, were decreased at 14 doa and tended to be lower at 40 doa in Hy-D fed birds than in commercial-fed broilers. Additionally, those birds that were injected with both doses of 25OHD_3_ in combination with the MDV experienced lower serum AGP concentrations than those in the MDV-alone and noninjected treatments. Previous studies have shown a positive relationship between chronic inflammatory responses with impaired broiler performance as well as SI histomorphology in response to the dietary (Chou et al., 2009; Fatemi, 2016) or in ovo (Fatemi et al., 2021d; Fatemi et al., 2022a) administration of 25OHD_3_. This may be related to an enhanced expression of genes that are involved with anti-inflammatory responses. An up-regulated expression of IL-10 has been shown to occur in response to dietary 25OHD_3_ in birds subjected to lipopolysaccharide (Morris and Selvaraj, 2014; Morris et al., 2014) or coccidial challenges (Morris et al., 2015; Shanmugasundaram et al., 2019). Furthermore, in comparison to noninjected and diluent-injected treatments, the expression of genes linked to anti-inflammatory responses increased in broilers that were injected with 2.4 μg of 25OHD_3_. Therefore, it can be extrapolated that partial improvements in SI morphological variables could be due to a decrease in inflammation in response to both sources of 25OHD_3_ administered in combination with the MDV.

In this study, no significant main or interactive effects were observed for serum NO concentrations throughout the rearing period. Similarly, plasma NO concentration did not differ when broilers received an *in ovo* injection of medium to high (12 or 25 mg) doses of L-ascorbic acid, although the NO concentration was higher in 14-d-old male rather than female broiler hatchings when they were reared under commercial conditions (Mousstaaid et al., 2022b). In addition, serum NO concentrations did not differ in birds belonging to different D_3_ source treatments when they were reared on fresh or reused litter (Fatemi, 2016). However, when compared at 28 doa to diluent-injected or 2.4 µg D_3_-injected broilers, serum NO levels in those subjected to a coccidiosis infection were reduced in response to the in ovo injection of 2.4 µg of 25OHD_3_ (Fatemi et al., 2022a). This indicates that serum NO concentration is an appropriate inflammation indicator when birds experience severe chronic systemic inflammation. In light of those reports, it is suggested that birds in this study did not encounter a severe inflammatory challenge despite being reared on reused litter. However, mild changes in the indicators of inflammation could be linked to the dietary or in ovo 25OHD_3_ treatments.

In conclusion, in response to the in ovo administration of the MDV in combination with various levels of 25OHD_3_, the posthatch SI morphology and immunity of broilers were investigated. It was observed that when compared to noninjected and MDV-alone-injected treatment groups, both sources of 25OHD_3_ were effective in increasing the VL and VCR and in decreasing the CD in the mid to lower SI of broilers through the rearing period. Furthermore, in comparison to MDV-alone-injected and noninjected treatment controls, AGP, as an inflammation indicator, was reduced in birds belonging to the MDV + 25OHD_3_-1.2 or MDV + 25OHD_3_-2.4 treatment groups. In addition, birds fed Hy-D diets had a lower AGP at 14 doa and tended to have a lower AGP at 40 doa when compared to those fed commercial diets. However, the dietary source of 25OHD_3_ appears to be more potent towards improving the SI histomorphology and the inflammatory reaction of broilers throughout the rearing period. It is further suggested that the supplemental administration of 25OHD_3_ via in ovo injection or the diet can be used to improve the posthatch gut health of broilers that had received an in ovo injection of the MDV.

## DISCLOSURES

The authors declare no conflicts of interest.
